# Advanced Lung Cancer Patients’ Use of EGFR Tyrosine Kinase Inhibitors and Overall Survival: Real-World Evidence from Quebec, Canada

**DOI:** 10.3390/curroncol29110636

**Published:** 2022-10-26

**Authors:** Samia Qureshi, Gino Boily, Jim Boulanger, Kossi Thomas Golo, Aude-Christine Guédon, Camille Lehuédé, Ferdaous Roussafi, Catherine Truchon, Erin Strumpf

**Affiliations:** 1 Department of Epidemiology, Biostatistics and Occupational Health (EBOH), McGill University, Montreal, QC H3A 1G1, Canada; 2 Institut national d’excellence en santé et services sociaux (INESSS), Montreal, QC H3A 2S9, Canada; 3 Institut national d’excellence en santé et services sociaux (INESSS), Quebec City, QC G1V 4M3, Canada

**Keywords:** non-small cell lung cancer (NSCLC), metastatic cancer, advanced cancer, targeted therapies, epidermal growth factor receptor-tyrosine kinase inhibitors (EGFR-TKIs), real-world data, real-word evidence, survival, treatment utilization

## Abstract

EGFR tyrosine kinase inhibitors (EGFR-TKIs) are breakthrough palliative treatments for advanced lung cancer patients with tumors harboring mutations in the EGFR gene. Using healthcare administrative data, three cohorts were created to describe the use of three EGFR-TKIs that are publicly funded in Quebec for specific indications (i.e., 1st-line gefitinib, 1st-line afatinib, and post-EGFR-TKI osimertinib). The main objective was to compare overall survival (OS) among patients receiving these treatments to those in previous experimental and real-world studies. The patients who received EGFR-TKIs for indications of interest between 1 April 2001, and 31 March 2019 (or 31 March 2020, for post-EGFR-TKI osimertinib) were included to estimate the Kaplan-Meier-based median OS for each cohort. An extensive literature search was conducted to include comparable studies. For the gefitinib 1st-line (n = 457), the afatinib 1st-line (n = 80), and the post-EGFR-TKI osimertinib (n = 119) cohorts, we found a median OS (in months) of 18.9 (95%CI: 16.3–21.9), 26.6 (95%CI: 13.7-NE) and 19.9 (95%CI: 17.4-NE), respectively. Out of the 20 studies that we retained from the literature review and where comparisons were feasible, 17 (85%) had similar OS results, which further confirms the value of these breakthrough therapies in real-world clinical practice.

## 1. Introduction

With about half of all cases being diagnosed at stage IV [1], lung cancer is the deadliest cancer in Canada, including Quebec [2]. Patients diagnosed with lung cancer in Canada between 2015 and 2017 had a net survival rate of 22% [2]. Only 24% of all cases in Quebec diagnosed between 2014 to 2016 survived 5 years or more from any cause of death [3].

Patients with advanced cancer (i.e., locally advanced, or metastatic) with limited curative treatment options are typically given palliative treatment with chemotherapy, immunotherapy, or targeted therapy, the latter two being breakthrough therapies introduced in the past decade. Since 2011 in Quebec, oral EGFR tyrosine kinase inhibitors (EGFR-TKI) have been a standard targeted treatment for patients with tumors harboring EGFR-TKI sensitizing mutations, which include resistance mutations [4,5]. In Ontario, up to 20% of advanced patients with non-squamous, non-small-cell lung cancer have the most common EGFR-TKI sensitizing mutations (exon 19 deletions and exon 21-L858R) [6]. About 50–65% of patients progressing on first- and second-generation EGFR-TKIs develop an EGFR-TKI resistance mutation (T790M) that can be treated with third-generation EGFR-TKIs [7,8].

In Quebec, the Institut national d’excellence en santé et en services sociaux (INESSS) evaluates the evidence on new drugs, usually based on at least one randomized controlled trial (RCT), and provides recommendations to the Minister of Health and Social Services who regulates the drug formulary of Quebec’s universal public drug coverage program [9]. EGFR-TKIs are publicly covered in Quebec for specific indications (e.g., line of treatment, EGFR mutation status, etc…) and the clinical guidelines for treatment with these EGFR-TKIs are mainly based on benefits observed in RCTs in surrogate endpoints of overall survival (e.g., progression-free survival [PFS]) [5]. Despite the availability of mature data from these trials, some participants in the control arm received EGFR-TKIs following disease progression, which potentially masked overall survival (OS) benefits.

INESSS is exploring real-world data to assess the value of breakthrough therapies in Quebec’s clinical settings, given its potential for complementing evidence from small prospective studies and assessing the real-world value of interventions after their implementation into practice [10,11,12,13,14]. If results from real-world studies show smaller benefit than RCTs, review of the funding criteria or decision could be justified. This population-based retrospective study provides such evidence to policymakers and other stakeholders by drawing a portrait of advanced lung cancer patients who received EGFR-TKIs that are publicly covered in Quebec for specific indications and comparing estimates of OS in this population to those published in RCTs and real-world studies.

## 2. Materials and Methods

### 2.1. Study Design and Data

Our study included patients who had public coverage for prescriptions filled at community pharmacies in Quebec and were treated with EGFR-TKIs for indications included in Quebec’s drug formulary (Table 1): gefitinib or afatinib for 1st-line palliative treatment, or osimertinib as a second EGFR-TKI treatment following 1st-line palliative treatment with an EGFR-TKI. Since the public coverage of EGFR-TKIs requires a treating physician to confirm their patient’s status as advanced cancer with an activating EGFR mutation, payments of these drugs by the public insurer allowed us to infer this status for patients in our study. We did not investigate EGFR-TKIs that were (1) not in Quebec’s drug formulary (e.g., dacomitinib), (2) in Quebec’s drug formulary for indications included after our recruitment period (e.g., osimertinib for 1st-line treatment), and (3) in Quebec’s drug formulary without EGFR mutation status as an indication and reimbursement criterion (e.g., erlotinib for 2nd- or 3rd-line treatments).

Inpatient and outpatient care administrative data (i.e., hospitalization [MED-ECHO], physician billing [SMOD], drugs dispensed in community pharmacies [SMED], health insurance registry [FIPA]) that is managed by the Régie de l’assurance maladie du Québec (RAMQ) and the Ministère de la Santé et des Services sociaux (MSSS) were used to create three EGFR-TKI cohorts: (1) 1st-line gefitinib, (2) 1st-line afatinib, and (3) post-EGFR-TKI osimertinib. For all cohorts, we selected patients who had their first lung cancer diagnostic code in MED-ECHO or SMOD between 1 April 2001, and 31 March 2019, (lung cancer index date) [3] with at least one gefitinib, afatinib, or osimertinib prescription in the same period. We also included in the osimertinib cohort patients with at least one osimertinib prescription before 31 March 2020, after receiving gefitinib, afatinib, or erlotinib between 1 April 2001, and 31 March 2019. International nonproprietary names and drug identification numbers were used to identify EGFR-TKI prescriptions in SMED (Appendix A). All patients were followed until death (captured in FIPA), or 31 March 2020, whichever came first.

We then excluded from each cohort patients that did not receive an EGFR-TKI for the line of treatment of interest. Since we had limited information on the line of palliative treatment and chemotherapy history, we developed an algorithm to identify the line of therapy associated with patients’ 1st EGFR-TKI (Appendix B). From each cohort, we first excluded patients who were covered by a public drug insurance plan in FIPA for less than 90% of the period from 3 months before the 1st EGFR-TKI treatment to the end of follow-up (targeted treatment observation period). From the gefitinib and afatinib cohorts, we also excluded patients whose 1st EGFR-TKI was not gefitinib or afatinib, and for the osimertinib cohort, we excluded those whose 2nd EGFR-TKI was not osimertinib after receiving gefitinib, afatinib, or erlotinib. All patients identified by the algorithm as receiving their 1st EGFR-TKI other than 1st-line palliative treatment were then excluded to form the final cohorts.

This study was carried out at INESSS as part of a Health System Impact Fellowship (SQ) of the Canadian Institutes of Health Research. It was part of an initiative to promote the use of real-world data for health technology assessment in line with INESSS’s work on real-world evidence in its 2019–2022 Three-Year Business Plan which aims to “Assess the value of interventions in real care settings using medico-administrative data” [16]. INESSS is not responsible for the content of this publication, however, the results reported here were part of a larger INESSS project to study EGFR-TKIs in Quebec [17]. Access to de-identified data for this study was made possible through a tripartite agreement between the MSSS, the RAMQ, and INESSS [18]. Clinical experts were consulted to select relevant intervention codes and develop the algorithm used to identify line of treatment. A scientific advisory committee and INESSS’ Comité de l’évolution des pratiques en oncologie (CEPO), a panel of experts in cancer (hemato- and radio-oncologists, surgeons and pharmacists), were consulted throughout the study [17].

### 2.2. Outcomes

For each cohort, we reported the annual number of new users and other estimates from the distributions of patient characteristics, total days of the EGFR-TKI supplied, and OS.

The date of the 1st EGFR-TKI prescription fill was used to count the number of new users by fiscal year (April to March) and calculate patients’ age. The age and sex distributions in each cohort were compared to distributions of a previous population-level lung cancer cohort created by INESSS with Quebec’s health administrative databases [3]. For the post-EGFR-TKI osimertinib cohort, we reported the EGFR-TKI that was also received as 1st-line treatment. The total number of days’ supply of EGFR-TKI per patient was obtained by adding the treatment duration (in days) of each EGFR-TKI prescription reimbursed by the RAMQ.

Patients’ overall survival time was calculated as months elapsed between patients’ first EGFR-TKI prescription of interest and the date of death from any cause. Administrative censoring occurred on 31 March 2020. Patients’ follow-up in the 1st-line gefitinib and afatinib cohorts was also censored at the time of receiving osimertinib, if applicable; osimertinib increases OS relative to standard chemotherapy when given as a subsequent line after another EGFR-TKI [19,20]. Censoring at the time of receiving osimertinib was applied to compare our survival results to studies in which patients did not receive osimertinib as a subsequent therapy, including studies submitted to INESSS for evaluation of gefitinib and afatinib.

### 2.3. Statistical Analyses

Frequencies and proportions, means and standard deviations, or medians and ranges (minimum to maximum), were reported for distributions of age, sex, the 1st-line EGFR-TKI drug, and new users. Boxplots were used to display the distribution of total days’ supply. Median follow-up time was estimated with the reverse Kaplan-Meier method in the *prodlim* package in R [21], and the *survival* package was used to plot Kaplan-Meier survival curves and estimate median overall survival times [22].

### 2.4. Comparison of Overall Survival

Our study could not include a comparator group of patients with EGFR mutated tumors who were not treated with EGFR-TKIs since we did not have information on mutation status, and more importantly, in the real-world clinical setting, it would be unethical to withhold EGFR-TKI treatment in the presence of an activating EGFR mutation. Therefore, we relied on the indirect comparison of the median OS in our cohorts to that of cohorts with similar EGFR-TKI use in previous studies. In May 2021, we conducted an extensive literature review to include all published experimental trials and real-world studies that reported OS estimates related to the three EGFR-TKIs for the indications of interest (Appendix C). The studies submitted to INESSS for evaluation of these treatments that reported estimates of median OS were automatically included in our review [20,23,24,25,26,27,28].

The similarity between our results and those of other studies (point estimates and 95% confidence intervals) was assessed with the degree of overlap that took into consideration the absolute difference between point estimates and the width of the confidence intervals, but without any formal statistical tests.

## 3. Results

**Figure 1:**
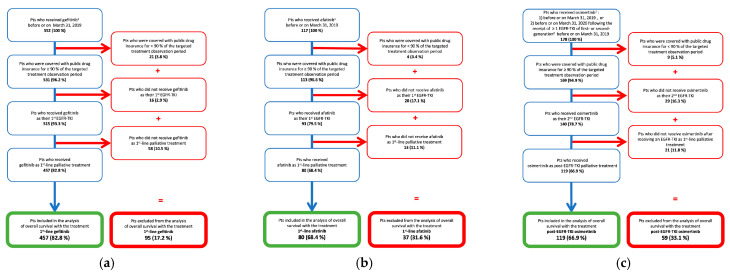
Flowchart of study populations in EGFR-TKI cohorts: (**a**) 1st-line gefitinib cohort; (**b**) 1st-line afatinib cohort; (**c**) Post-EGFR-TKI osimertinib cohort. Abbreviations: Pts: Patients. ^1^ End of follow-up: 31 March 2020. ^2^ Gefitinib, afatinib, or erlotinib.

**Figure 2:**
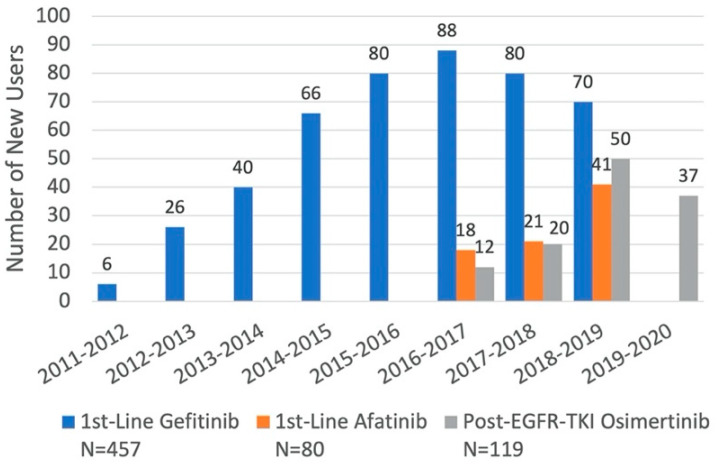
Distribution of New Users of Each EGFR-TKI for Specific Indications, by Fiscal Year.

Among 552 patients who received gefitinib, 457 (82.8%) were included in the 1st-line gefitinib cohort. Out of 117 patients receiving afatinib, 80 (68.4%) were included in the 1st-line afatinib cohort. Similarly, of 178 patients who received osimertinib, 119 (66.9%) were included in the post-EGFR-TKI osimertinib cohort (Figure 1). New users of 1st-line palliative treatments with gefitinib or afatinib started to accrue in the same year as their listing in Quebec’s drug formulary in November 2011 and May 2016, respectively. In contrast, 32 patients in the osimertinib cohort received the drug in the 2 years before it was listed on the formulary in November 2018, when the highest numbers of users were observed (Figure 2 and Table 1).

Compared to lung cancer patients in Quebec, there was a larger proportion of women in all three cohorts (60–71% vs. 49%) (Table 2 and Appendix D). The median age for the gefitinib cohort was the same as the overall cohort (71 years), whereas the median age for the afatinib cohort was lower (68 years) and that for the osimertinib cohort was higher (72 years). There was a much smaller portion of patients that were 80 years and over in the afatinib cohort in comparison to the overall cohort (7.5% vs 20.6%).

**Figure 3:**
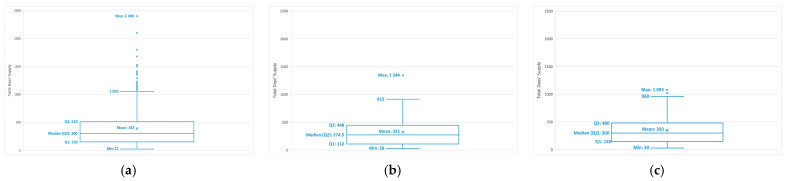
Distribution of total days’ supply: (**a**) 1st-line gefitinib cohort (N = 457); (**b**) 1st-line afatinib cohort (N = 80); (**c**) Post-EGFR-TKI osimertinib cohort (N = 119). Abbreviations: min: minimum; Q1: quartile 1; Q2: quartile 2 or median; Q3: quartile 3; max: maximum. The lower limit of the boxplot mustache represents the minimum, whereas the superior limit is equal to 1.5 times the interquartile range (Q3 minus Q1) plus Q3.

The median total days’ supply was 300 days (9.9 months) for gefitinib and osimertinib, and 274.5 days (9.0 months) for afatinib (Figure 3). Approximately 5% of patients had a total days’ supply of gefitinib that was greater than 3 years. Only 1 patient received a supply of afatinib for more than 3 years, while no one received a supply of osimertinib for this length of time.

**Figure 4:**
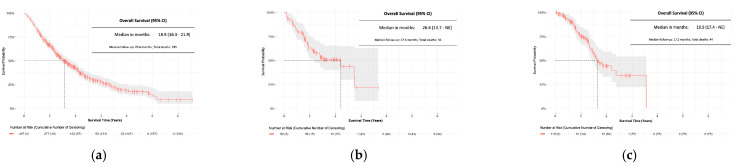
Overall survival: (**a**) 1st-line gefitinib cohort (N = 457); (**b**) 1st-line afatinib cohort (N = 80); (**c**) Post-EGFR-TKI osimertinib cohort (N = 119). Abbreviations: 95% CI: 95% confidence interval. The grey area around the curves represents the 95% confidence intervals.

With a median follow-up of 29.6 months in the gefitinib cohort, we observed 295 (65%) deaths and a median OS of 18.9 months (95%CI: 16.3–21.9) (Figure 4). The afatinib cohort had a median follow-up of 17.6 months, 36 (45%) deaths and a median OS of 26.6 months (95%CI: 13.7-NE). With a median of 17.3 months of follow-up after receiving osimertinib in the post-EGFR-TKI osimertinib cohort, we observed 44 (38%) deaths and median OS was 19.9 months (95%CI: 17.4-NE). The upper bounds of the 95%CI for median OS in the afatinib and osimertinib groups were non-evaluable (NE) due to the immaturity of the data (i.e., less than 50% of patients in the cohort had the outcome).

**Figure 5:**
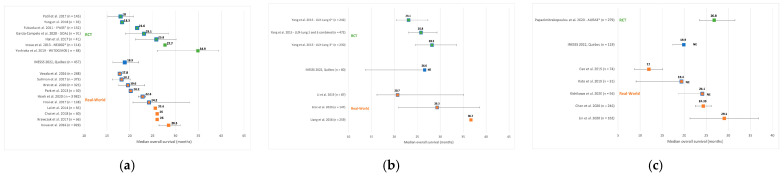
Comparison of overall survival estimates with RCTs and real-world studies: (**a**) 1st-line gefitinib; (**b**) 1st-line afatinib; (**c**) Post-EGFR-TKI osimertinib cohort. * Studies submitted to INESSS for evaluation. Abbreviations: RCT: randomized controlled trial; NE: upper bound of 95% confidence interval is non-evaluable. The mustache around each estimate represents the 95% confidence interval. The orange or green square with a blue outline represents a median that is considered similar to Quebec’s median.

From 2565 articles identified, we selected 10 RCTs (9 articles) and 17 real-world studies (18 articles) for indirect comparison with our OS results (Appendix C). We retained 7 RCTs [26,27,29,30,31,32,33] and 10 real-world studies [34,35,36,37,38,39,40,41,42,43] on gefitinib as 1st-line treatment (Figure 5). Among these studies, 5 RCTs and 6 real-world studies had median OS estimates similar to ours, 1 RCT [30] and 1 real-world study [40] had results superior to ours. We were limited in making comparisons with the estimates from other studies, which did not overlap our results and lacked confidence intervals. For afatinib as 1st-line treatment we retained 2 RCTs [28], and 1 study combining these RCTs [28], and 3 real-word studies [34,44,45]. All studies had similar results to ours, except for 1 real-world study for which we were limited in making a comparison due to a lack of overlap of results and the non-evaluable upper bound for our estimate’s 95%CI. We retained only 1 RCT [20] and 5 real-world studies [46,47,48,49,50] on osimertinib as a post-EGFR-TKI treatment. One real-world study [49] had a median inferior to ours and two others [47,50] had results similar to ours. Due to a lack of overlap of results and the non-evaluable upper bound of our estimate’s 95%CI, our comparisons with the other 2 real-world studies [46,48] were limited. Similarly, we were limited in the comparison with the single RCT, which had a longer median OS.

## 4. Discussion

In our study, the annual number of new users of gefitinib always exceeded the number of new patients using the second-generation EGFR-TKI afatinib as a 1st-line treatment. However, a decline in the use of gefitinib relative to afatinib was noted in the years following the introduction of afatinib in 2016. In contrast with other EGFR-TKIs, the use of osimertinib began before it was listed in Quebec’s drug formulary, which most likely occurred through the “exceptional patient program” that provides coverage for drugs that are not listed in Quebec’s drug formulary under exceptional circumstances. These trends indicate physicians’ proactiveness in integrating newer generations of EGFR-TKIs into clinical practice.

The high proportion of women (60–71%) we observed in all three of our cohorts is concordant with a previous study reporting that 57% of EGFR mutations in lung cancer tumours are found in women in Canada [6]. Our results are also concordant with the studies we selected from our literature review and one recent single-center study in Quebec that reported 71% of its 1st-line EGFR-TKI users being women [51]. Knowledge of a higher EGFR mutation rate in women could also drive a higher testing rate in women, as seen previously in Canada [52], and further contribute to the higher rates of EGFR-TKI use in women.

In general, all our cohorts included patients above the age of 80 years. Older patients may be less likely to receive cytotoxic chemotherapy due to a lack of clinical data on tolerability, but also due to clinical experience with the elderly who show more complications. In contrast, EGFR-TKIs have better tolerability than chemotherapy [19,32,33,53,54,55]. Furthermore, in RCTs on EGFR-TKIs, patients older than 80 years (maximum of 89 years) have been included [19,29,31,56,57], and studies on patients of 75 years or older, have reported median OS estimates similar to those of the overall population (19–35.2 months) [58,59,60,61,62,63]. However, we noted that patients in the afatinib cohort, compared to the gefitinib cohort, had a lower proportion of patients of 80 years and older (7.5% vs. 17.5%). Physicians may be hesitant in prescribing afatinib for the elderly due to its higher toxicity profile in comparison to gefitinib [56]. The trend of fewer older patients using afatinib in comparison to gefitinib has also been observed in other real-world studies [34,44,64].

When daily treatment is continuous until disease progression, estimates of total days’ supply should resemble time to treatment discontinuation, which is a proxy for PFS [65]. Indeed, the total days’ supply of EGFR-TKI in our cohorts were close to the PFS reported in the RCTs we selected from our literature review, and in one recent single-center study in Quebec. The studies reported a PFS of 8.4–11.9 months (median 10.4) for gefitinib as 1st-line treatment [27,29,31,32,33,51,53,54], and a PFS of 10.1 months for osimertinib as a post-EGFR-TKI treatment [20], while we found medians of total days’ supply of 9.9 months for both treatments. Two RCTs reported a PFS of 11.0–11.1 months for afatinib as a 1st-line treatment [28], while we found a total days’ supply of 9 months. Total days’ supply and PFS may not be equivalent when daily treatment is paused or discontinued for reasons other than progression, such as adverse events. Therefore, the small difference between both parameters that we observed for afatinib as a 1st-line treatment may have been led by its higher toxicity profile.

When indirect comparisons with our OS estimates were possible, we found 3 RCTs submitted to INESSS for evaluation of gefitinib and afatinib (IPASS, LUX-Lung 3, and LUX-Lung 6) to have results similar to ours. Among the 12 other RCT and real-world studies on gefitinib that we were able to indirectly compare with the current study, 10 studies had similar results. The 2 real-world studies on afatinib that we were able to compare with our study also had similar results. Similarly, among the 3 real-world studies on osimertinib that we were able to compare with, 2 had similar results. There were 7 out of 27 studies that reported longer median OS estimates than those in the current study, but these comparisons were limited because of a lack of overlap of results due to unreported or non-evaluable confidence intervals. Despite the uncertainty in these comparisons, it is likely that the OS gap between the RCT of osimertinib (AURA3) and the current study is real. Contrary to AURA3, patients in our post-EGFR-TKI osimertinib cohort may have received chemotherapy in between their 1st-line treatment with an EGFR-TKI and osimertinib as a second EGFR-TKI, which may have reduced the length of survival at the time of receiving osimertinib.

Based on the indirect comparisons, we conclude that the real-world estimates of OS in Quebec related to the use of the three EGFR-TKIs for specific indications are not different from most published studies that we selected from our literature review. This is further supported by one recent population-level study in Ontario that reported results similar to ours: a median OS of 21.6 months (95%CI: 19.3–23.3) for 1st-line gefitinib, and a median OS of 31.0 months (95%CI: 23.4–42.1) for 1st-line afatinib [64].

The limitations of this study include that we could not directly verify whether patients had a non-small cell type histology and an activating EGFR mutation. However, public coverage of EGFR-TKIs is conditional on these characteristics, making it unlikely for patients receiving public coverage of these medicines not to have them. The indirect comparisons in this study are subject to confounding by multiple factors such as age, smoking status, the type of EGFR mutation, and palliative treatments following EGFR-TKIs. For example, we did not include patients that had received EGFR-TKIs through private drug insurance, which likely rendered our cohorts slightly older than the ones in the RCTs submitted to INESSS for evaluations. However, we estimate the proportion of patients with private drug insurance among all EGFR-TKI users to be low since about 70% of lung cancer patients in Quebec are diagnosed at age 65 or older, when 90% of Quebec residents are registered for public drug coverage [3,66,67]. Finally, the survival estimates for the afatinib and osimertinib cohorts are less precise due to data immaturity, and the indirect comparisons of results from these cohorts with other studies imply a greater level of uncertainty.

Our study also has strengths. To our knowledge, this is the first population-level study on the use of EGFR-TKIs for specific indications in a Canadian province where public drug insurance offers full coverage of oral cancer drugs [66]. The involvement of clinical experts and a scientific committee helped us generate real-world evidence on these breakthrough therapies with analyses of Quebec’s health administrative data that are grounded in clinical and scientific expertise. Our holistic analytical approach through an extensive literature review also produced more meaningful results.

## 5. Conclusions

RCTs have shown that EGFR-TKIs are breakthrough therapies for advanced lung cancer patients. EGFR-TKIs have been included in Quebec’s drug formulary for specific indications, mainly based on the efficacy observed in surrogate endpoints of OS. Our study further confirms the value of these treatments in real-world clinical care, with OS rates in Quebec similar to those reported in most real-world studies and RCTs, including most RCTs used in the evaluation of these treatments for public drug coverage. Future studies should re-evaluate EGFR-TKIs with mature and richer data and, given their potential real-world value, further investigate inequities in access to these treatments.

## Figures and Tables

**Table 1:** Indications for gefitinib, afatinib, and osimertinib included in the drug formulary of Quebec’s universal public drug coverage program [15].

EGFR-TKI	Line of Palliative Treatment	Indication and Coverage Criteria	Date Added to Formulary
Gefitinib	1st line	1st-line treatment of patients with locally advanced or metastatic NSCLC, with an activating EGFR mutation, and with an ECOG performance status of 0 to 2.	November 2011
Afatinib	1st line	In monotherapy for the 1st-line treatment of patients with metastatic NSCLC, with an activating EGFR mutation, and with an ECOG performance status of 0 or 1.	May 2016
Osimertinib	2nd line or more	Treatment of locally advanced, unresectable, or metastatic NSCLC, with the T790M EGFR mutation in patients: Whose disease progressed during or following treatment with an EGFR-TKI Whose ECOG performance status is 0 or 1.	November 2018
	1st line ^1^	1st-line treatment of people with locally advanced, unresectable, or metastatic NSCLC, with an activating EGFR mutation, and with an ECOG performance status of 0 to 1.	December 2018

Abbreviations: NSCLC: non-small-cell lung cancer; ECOG: Eastern Cooperative Oncology Group. ^1^ EGFR-TKI for this indication was not studied.

**Table 2:** Characteristics of patients in EGR-TKI cohorts.

Characteristics	1st-Line Gefitinib Cohort ^1^		1st-Line Afatinib Cohort ^1^		Post-EGFR-TKI Osimertinib Cohort ^1^		Lung Cancer Cohort 2016 ^2^	
	n	(%)	n	(%)	n	(%)	n	(%)
**Sex**								
Female	313	(68.5)	48	(60.0)	84	(70.6)	4803	(49.3)
Male	144	(31.5)	32	(40.0)	35	(29.4)	4936	(50.6)
**Age in years**								
Median (range) ^3^	70.6	(34; 93)	68.4	(22; 93)	72.3	(24; 92)	70.7	(NR)
<50 years	15	(3.3)	4	(5.0)	5	(4.2)	249	(2.6)
50–64 years	91	(19.9)	24	(30.0)	26	(21.8)	2716	(27.9)
65–79 years	271	(59.3)	46	(57.5)	64	(53.8)	4762	(48.8)
≥80	80	(17.5)	6	(7.5)	24	(20.2)	2012	(20.6)
**1st-line EGFR-TKI**								
Gefitinib	457	(100)	-		92	(77.5)	-	
Afatinib	-		80	(100)	25	(21.0)	-	
Erlotinib	-		-		2	(1.7)	-	
**Total**	457		80		119		9752	

Abbreviations: NR: not reported. ^1^ Age measured at the start of the EGFR-TKI of interest. ^2^ Quebec lung cancer cohort developed by INESSS, in which age was measured at the diagnosis date [3]. ^3^ Range: minimum; maximum.

**Figure A1:**
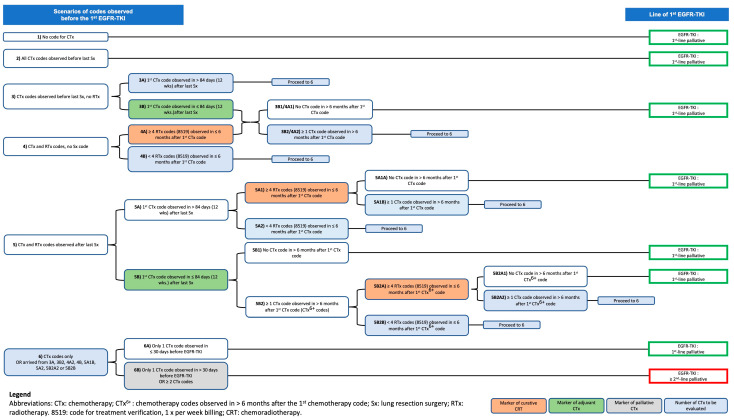
Classification of patients according to the line of treatment with 1st EGFR-TKI.

**Figure A2:**
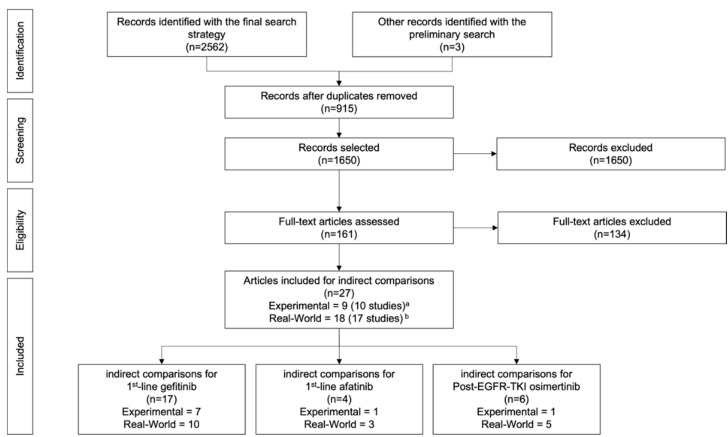
Flow Diagram of the Article Selection Process. ^a^ 1 article [28] included 2 randomized controlled trials (i.e., 2 studies) on afatinib as 1st-line treatment. ^b^ 1 article [34], which included 1 real-world study, was counted twice since it was used in the indirect comparisons of EGFR-TKIs for 2 indications: 1st-line gefitinib treatment and 1st-line afatinib treatment.

## Data Availability

Restrictions apply to the availability of these data. Data are available to INESSS through RAMQ servers due to the tripartite agreement.

## Appendix A. Identification of EGFR-TKIs in Health Administrative Databases

**Table A1:** International Non-Proprietary Name Codes and Drug Identification Numbers of EGFR-TKIs.

International Non-Proprietary Name (INN)	RAMQ’s Code for INN	Drug Identification Number (DIN)
Gefitinib	47482	02468050 02248676 02487748 02491796
Afatinib	47998 48096	02415666 02415674 02415682
Osimertinib	48112	02456214 02456222
Erlotinib	47563	02461862 02461870 02461889 02483912 02483920 02483939 02454386 02454394 02269007 02269015 02269023 02377691 02377705 02377713

## Appendix B. Algorithm to Identify the Line of Therapy Linked to Patients’ 1st EGFR-TKI Treatment

We first excluded patients who had a public drug insurance plan for less than 90% of the time covering 3 months before their 1st EGFR-TKI treatment to the time of death or 31 March 2020, whichever came first (i.e., targeted treatment observation period). This minimized the possibility of missing information on targeted treatments that patients may have received through private coverage. We then established the chronological sequence of all publicly reimbursed targeted therapies received between 1 April 2001, and 31 March 2020, including anaplastic lymphoma tyrosine kinase inhibitors (ALK-TKI). A new line of treatment was considered every time a patient switched targeted therapy drugs, with the possibility of an EGFR-TKI drug being assigned multiple lines.

History of cancer-related treatments (i.e., lung surgery, chemotherapy supervision, and radiotherapy) received between patients’ diagnosis date [3] and their 1st EGFR-TKI was retrieved from intervention codes in hospitalization and physician billing databases: MED-ECHO and SMOD. Patients were first grouped into 6 treatment code scenarios, and their 1st EGFR-TKI was further classified as 1st-line palliative treatment or 2nd-line or more of palliative treatment based on the previous use of chemotherapy and its context (curative versus palliative). We considered neoadjuvant chemotherapy, adjuvant chemotherapy, and chemoradiation as curative intent treatments, and, in consultation with medical experts, rules were created to identify these chemotherapies. Patients’ 1st EGFR-TKIs were assigned 1st-line treatment only in the absence of palliative chemotherapy.

Table A2 and Figure A1 further describe the steps to identify the line of treatment of patients’ 1st EGFR-TKI treatment. Table A1 (Appendix A) and Table A3 (Appendix B) provide international non-proprietary names and drug identification numbers used to identify targeted treatments in SMED and Table A4 provides intervention codes used to identify cancer-related treatments in SMOD and MED-ECHO.

More details on this algorithm can be found in a previous report [17].

**Table A2:** Treatment Scenarios and Rules to Identify Chemotherapy Context.

**1.** **No chemotherapy codes**	When no chemotherapy code is found prior the 1st EGFR-TKI, the latter is considered as 1st-line palliative treatment.
**2.** **All chemotherapy codes found before patient’s last lung resection surgery**	Since lung resection is considered a curative treatment (except in some cases), chemotherapy that is received prior to this surgery is also considered a curative treatment and it usually corresponds to neoadjuvant chemotherapy. The EGFR-TKI treatment that follow this chemotherapy is considered 1st-line palliative treatment.
**3.** **Chemotherapy codes found after patient’s last lung resection surgery and no radiotherapy codes**	Chemotherapy that comes after lung resection can correspond to adjuvant chemotherapy, which is given with curative intent if certain temporal conditions are met. Firstly, the chemotherapy should be given within 6 weeks of surgery. In practice, there may be a longer delay, therefore, we selected a more realistic margin of 12 weeks (≤84 days) that will allow the identification of most patients that received adjuvant chemotherapy (experts’ opinion). Secondly, since this type of chemotherapy usually involves 4 treatment cycles (4 months) [68], it should occur within a limited timeframe within patient’s treatment trajectory. We selected a margin of 6 months after the 1st chemotherapy code to account for potential treatment delays. When the 1st temporal condition is met (chemotherapy ≤ 12 weeks or 84 days after surgery), the chemotherapy is considered as adjuvant (curative intent; scenario 3B). If no additional chemotherapy code is found after 6 months, the patient is considered to receive no palliative chemotherapy and their 1st EGFR-TKI is considered as 1st-line palliative treatment (scenario 3B1). If ≥1 chemotherapy code is found after the 6 months period (scenario 3B2), the patient is moved into scenario 6 (see scenario 6). When the 1st chemotherapy occurs >12 weeks after surgery (scenario 3A), the patient is moved into scenario 6 (see scenario 6).
**4.** **Chemotherapy and radiotherapy codes, and no lung resection surgery codes**	The physician billing code 8519 corresponds to treatment verification of an irradiated site (under treatment), and, according to experts that were consulted, it is systematically billed on a weekly basis throughout the treatment. Therefore, this code allows the estimation of the duration of radiotherapy. In the absence of a lung surgery, a combination of chemotherapy and radiation therapy can be administered with curative intent (chemoradiation) or in palliative intent (chemotherapy + palliative radiotherapy). The difference between these two types of treatments is that curative chemoradiation involves a higher dose of radiotherapy (generally 60-66 Gy for 6–7 weeks according to standard fractionation) [4]. Identifying ≥4 weeks of radiotherapy (≥4 × code 8519) can be used as a marker of curative treatment, whereas the presence of ≤3 weeks of radiotherapy corresponds to a palliative treatment (experts’ opinion). If the radiotherapy is considered curative (scenario 4A), the algorithm verifies if all chemotherapies occur within 6 months. If no chemotherapy is found beyond 6 months of the 1st chemotherapy, the patient is considered to receive no palliative chemotherapy and their 1st EGFR-TKI is considered as 1st-line palliative treatment (scenario 4A1). If ≥1 chemotherapy code is found after the 6 months of the 1st chemotherapy (scenario 4A2), the patient is moved into scenario 6 (see scenario 6).
**5.** **Chemotherapy and radiotherapy codes found after patient’s last lung resection surgery**	When chemotherapy and radiotherapy codes are observed after lung surgery, they could be related to (1) adjuvant/post-operative chemotherapy and radiotherapy treatments with curative intent, (2) chemotherapy and radiotherapy treatments with palliative intent, or (3) salvage chemoradiotherapy with curative intent. If the 1st chemotherapy code occurs in ≤12 weeks of the most recent lung resection (scenario 5B), it is considered as a curative treatment, irrespective of radiotherapy codes that may be present. If no chemotherapy is observed beyond 6 months after the 1st chemotherapy, the patient is considered to receive no palliative chemotherapy and their 1st EGFR-TKI is considered as 1st-line palliative treatment (scenario 5B1). If ≥1 chemotherapy code is found beyond 6 months of the 1st chemotherapy (scenario 5B2), the patient is assessed for receiving salvage chemoradiotherapy (curative) with the same steps as in scenario 4 (scenario 5B2A = 4A and scenario 5B2B = 4B). If the 1st chemotherapy code occurs in >12 weeks of the most recent lung resection (scenario 5A), the patient is assessed for receiving salvage chemoradiotherapy (curative) with the same steps as in scenario 4 (scenario 5A1 = 4A and scenario 5A2 = 4B).
**6.** **Chemotherapy codes only OR other scenarios that mention “proceed to 6”**	When chemotherapy is the only treatment observed, it always administered with palliative intent. Patients with this chemotherapy are considered to receive their 1st EGFR-TKI as 2nd-line or more of palliative treatment. An exception to this rule is when only 1 chemotherapy code is found which is within 30 days prior to the 1st EGFR-TKI, the latter is considered as 1st-line palliative treatment (scenario 6A). According to the experts we consulted, it is possible in clinic that some patients received a chemotherapy as an immediate treatment due to long delays in receiving mutation test results and patients pressing health state. In this case, the experts recommended that patients’ 1st EGFR-TKI be considered as a 1st-line palliative treatment

**Table A3:** International Non-Proprietary Names and Drug Identification Numbers of ALK-TKIs.

International Non-Proprietary Name (INN)	RAMQ’s Code for INN	Drug Identification Number (DIN)
Crizotinib	47913	02384256 02384264
Alectinib	48129	02458136
Céritinib	48049	02436779

**Table A4:** Cancer-Related Intervention Codes in SMOD and MED-ECHO.

A. Physician billing codes for fee-for-service renumeration	
A1. Chemotherapy (and Immunotherapy) ^1^	
734	Supervision de l’administration de chimiothérapie
15272	(Forfait pour Clinique de chimiothérapie) Suivi et administration, le cas échéant, des traitements de chimiothérapie intraveineuse aux patients atteints d’un cancer et dont le chirurgien général a la charge. NOTE: Cet acte est accordé pour les activités réalisées au sein d’une clinique spécifique d’oncologie, pour un minimum de 5 patients par demi-journée. NOTE: Maximum quatre fois par médecin, par période de quatorze jours. NOTE: Aucun procédé diagnostique et thérapeutique, aucune visite ni aucune chirurgie ne peut être facturé avec le code 15272.
15403	(Forfait pour Clinique de chimiothérapie) Suivi et administration, le cas échéant, des traitements de chimiothérapie intraveineuse aux patients atteints d’un cancer et dont le médecin spécialiste en médecine interne a la charge. NOTE: Cet acte est accordé pour les activités réalisées au sein d’une clinique spécifique d’oncologie, pour un minimum de 5 patients par demi-journée. NOTE: Maximum quatre fois, par médecin, par période de quatorze jours. NOTE: Aucun procédé diagnostique et thérapeutique, aucune visite ni aucune chirurgie ne peut être facturé avec le code 15403.
152	Perfusion intraveineuse de gammaglobuline, incluant la surveillance
**A2. Radiotherapy ^2^**	
8503	Injection intraveineuse de substance de contraste, supplément (Planification du traitement par radiations à l’aide de la tomodensitométrie—8553)
8504	Radiothérapie avec modulation d’intensité par planification inverse NOTE: Le code 08504 et le code 08564 sont mutuellement exclusifs.
8511	Évaluation et ajustement de la configuration des champs de radiation et de la collimation
8512	Soins médicaux à visée palliative, prodigués par un médecin spécialiste en radiooncologie, par site anatomique
8518	Vérification simulée de localisation à partir de documents radiologiques
**8519**	Vérification sous thérapie de site d’irradiation à partir de documents radiologiques. Maximum une fois par semaine, du lundi au dimanche, par patient, par site anatomique
8520	Étude de la dosimétrie à l’ordinateur (radiothérapie transcutanée)
8553	Planification du traitement par radiations à l’aide de la tomodensitométrie
8554	Irradiation stéréotaxique, incluant la planification et les séances de traitement, par site tumoral. Maximum 1 fois par patient, par site anatomique, par mois. Maximum 6 fois par patient à vie pour tous sites anatomiques. NOTE: Le code 08554 ne peut être facturé avec les codes 08511, 08518, 08520 et 08553 à la même séance.
8564	Radiothérapie avec modulation d’intensité
8565	Radio-oncologie/Fusion d’images
15465	Présentation à un ou plusieurs radiooncologues du dossier d’un patient avec évaluation formelle du plan de traitement élaboré, avec note au dossier. (centre hospitalier) NOTE: Le code 15465 n’est facturable que par le radiooncologue présentateur, et ce, par site anatomique.
20158	Planification du traitement par radiations avec imagerie multimodalité. NOTE: Le code 20158 ne peut être facturé avec le code 08565 à la même séance.
20159	Planification d’un site de réirradiation comportant un risque de chevauchement de champ d’irradiation utilisé dans le passé. NOTE: Le code 20159 ne peut être facturé plus d’une fois par patient, par trimestre.
20160	Planification du traitement par radiations à l’aide de la tomodensitométrie en 4D incluant la synchronisation respiratoire, le cas échéant. NOTE: Le code 20160 ne peut être facturé avec le code 20158, si effectué quinze jours avant ou après. NOTE: Le code 20160 ne peut être facturé avec le code 08565, le même jour.
20181	Bronchoscopie—avec insertion de marqueurs (grains d’or) au niveau pulmonaire (transbronchique) pour radiothérapie stéréotaxique, supplément
8507	Planification du traitement par radiations lésions non cutanées—plus de 30 min mais moins de 45 min, supplément
8508	Planification du traitement par radiations lésions non cutanées—45 min ou plus, supplément
8509	Planification du traitement par radiations à l’aide de la tomodensitométrie—plus de 45 min, supplément
**A3. Surgery—Lung Resection**	
3078	avec angioplastie, supplément (3125)
3079	Pneumonectomie, réintervention plus de 30 jours après l’intervention initiale, supplément
3122	Résection cunéiforme (Wedge)
3124	Segmentectomie simple incluant bronches et artère segmentaire
3125	Lobectomie simple avec ou sans évidement ganglionnaire
3126	segmentectomie additionnelle, supplément (3125)
3127	lobectomie moyenne (côté droit), supplément (3125)
3128	avec résection en manchon d’une bronche, supplément (3125)
3129	avec bronchoplastie, supplément (3125)
3130	résection de paroi thoracique, sans reconstruction, supplément (3125)
3131	résection de paroi thoracique, avec reconstruction prosthétique, tout type, supplément (3125)
3132	Lobectomie avec ou sans évidement ganglionnaire incluant résection de la paroi, pour tumeur de Pancoast
3133	Pneumonectomie simple avec ou sans évidement ganglionnaire
3134	Pneumonectomie simple—Péricardectomie (résection intrapéricardique), supplément
3135	Pneumonectomie simple avec résection de paroi thoracique sans reconstruction, supplément
3136	Pneumonectomie simple avec résection de paroi thoracique avec reconstruction, supplément
3137	Pneumonectomie simple avec résection de l’éperon trachéal incluant la réparation, supplément
3139	Réintervention pour lobectomie plus de 30 jours après l’intervention initiale, supplément
3140	Excision—chaque résection additionnelle (maximum 3), supplément (3122)
3162	pneumonectomie complémentaire si envahissement de la marge de résection, supplément (3125)
**B.** **Canadian classification of health interventions (CCI) codes**	
**B1. Chemotherapy**	
1ZZ35CAM0	Pharmacothérapie, corps entier, agents anticancéreux et immunomodulateurs approche par voie naturelle (orale) utilisation d’agents anticancéreux SAI
1ZZ35CAM1	Pharmacothérapie, corps entier, agents anticancéreux et immunomodulateurs approche par voie naturelle (orale) utilisation d’un agent alcoylant
1ZZ35CAM2	Pharmacothérapie, corps entier, agents anticancéreux et immunomodulateurs approche par voie naturelle (orale) utilisation d’agents antimétabolites
1ZZ35CAM3	Pharmacothérapie, corps entier, agents anticancéreux et immunomodulateurs approche par voie naturelle (orale) utilisation d’agents alcaloïdes et d’autres produits naturels
1ZZ35CAM4	Pharmacothérapie, corps entier, agents anticancéreux et immunomodulateurs approche par voie naturelle (orale) utilisation d’agents antibiotiques cytotoxiques et de substances similaires
1ZZ35CAM5	Pharmacothérapie, corps entier, agents anticancéreux et immunomodulateurs approche par voie naturelle (orale) utilisation d’autres agents anticancéreux
1ZZ35CAM7	Pharmacothérapie, corps entier, agents anticancéreux et immunomodulateurs approche par voie naturelle (orale) utilisation d’agents immunostimulants
1ZZ35CAM8	Pharmacothérapie, corps entier, agents anticancéreux et immunomodulateurs approche par voie naturelle (orale) utilisation d’agents immunosuppresseurs
1ZZ35CAM9	Pharmacothérapie, corps entier, agents anticancéreux et immunomodulateurs approche par voie naturelle (orale) utilisation d’agents anticancéreux combinés [multiples]
1ZZ35HAM0	Pharmacothérapie, corps entier, agents anticancéreux et immunomodulateurs approche percutanée [intramusculaire, intraveineuse, souscutanée, intradermique] utilisation d’agents anticancéreux SAI
1ZZ35HAM1	Pharmacothérapie, corps entier, agents anticancéreux et immunomodulateurs approche percutanée [intramusculaire, intraveineuse, souscutanée, intradermique] utilisation d’un agent alcoylant
1ZZ35HAM2	Pharmacothérapie, corps entier, agents anticancéreux et immunomodulateurs approche percutanée [intramusculaire, intraveineuse, souscutanée, intradermique] utilisation d’agents antimétabolites
1ZZ35HAM3	Pharmacothérapie, corps entier, agents anticancéreux et immunomodulateurs approche percutanée [intramusculaire, intraveineuse, souscutanée, intradermique] utilisation d’agents alcaloïdes et d’autres produits naturels
1ZZ35HAM4	Pharmacothérapie, corps entier, agents anticancéreux et immunomodulateurs approche percutanée [intramusculaire, intraveineuse, souscutanée, intradermique] utilisation d’agents antibiotiques cytotoxiques et de substances similaires
1ZZ35HAM5	Pharmacothérapie, corps entier, agents anticancéreux et immunomodulateurs approche percutanée [intramusculaire, intraveineuse, souscutanée, intradermique] utilisation d’autres agents anticancéreux
1ZZ35HAM7	Pharmacothérapie, corps entier, agents anticancéreux et immunomodulateurs approche percutanée [intramusculaire, intraveineuse, souscutanée, intradermique] utilisation d’agents immunostimulants
1ZZ35HAM8	Pharmacothérapie, corps entier, agents anticancéreux et immunomodulateurs approche percutanée [intramusculaire, intraveineuse, souscutanée, intradermique] utilisation d’agents immunosuppresseurs
1ZZ35HAM9	Pharmacothérapie, corps entier, agents anticancéreux et immunomodulateurs approche percutanée [intramusculaire, intraveineuse, souscutanée, intradermique] utilisation d’agents anticancéreux combinés [multiples]
1ZZ35YAM0	Pharmacothérapie, corps entier, agents anticancéreux et immunomodulateurs voie NCA [transdermique, etc.] utilisation d’agents anticancéreux SAI
1ZZ35YAM2	Pharmacothérapie, corps entier, agents anticancéreux et immunomodulateurs voie NCA [transdermique, etc.] utilisation d’agents antimétabolites
1ZZ35YAM3	Pharmacothérapie, corps entier, agents anticancéreux et immunomodulateurs voie NCA [transdermique, etc.] utilisation d’agents alcaloïdes et d’autres produits naturels
1ZZ35YAM4	Pharmacothérapie, corps entier, agents anticancéreux et immunomodulateurs voie NCA [transdermique, etc.] utilisation d’agents antibiotiques cytotoxiques et de substances similaires
1ZZ35YAM5	Pharmacothérapie, corps entier, agents anticancéreux et immunomodulateurs voie NCA [transdermique, etc.] utilisation d’autres agents anticancéreux
1ZZ35YAM9	Pharmacothérapie, corps entier, agents anticancéreux et immunomodulateurs voie NCA [transdermique, etc.] utilisation d’agents anticancéreux combinés [multiples]
**B2. Radiotherapy ^2^**	
1GT27JA	Rayonnements, poumon NCA, faisceau externe
**B3. Surgery—Lung Resection**	
1GM80DA	Réparation, bronches NCA, avec technique d’apposition simple [par exemple, suture] approche endoscopique (percutanée)
1GM80DAXXE	Réparation, bronches NCA, avec lambeau local approche endoscopique (percutanée)
1GM80LA	Réparation, bronches NCA, avec technique d’apposition simple [par exemple, suture] approche ouverte
1GM80LAXXE	Réparation, bronches NCA, avec lambeau local approche ouverte
1GM80LAXXG	Réparation, bronches NCA, avec lambeau pédiculé à distance approche ouverte
1GM87DA	Excision partielle, bronches NCA, approche endoscopique (percutanée)
1GM87LA	Excision partielle, bronches NCA, approche ouverte
1GN92LA	Excision radicale avec reconstruction, éperon trachéal, approche ouverte
1GR87DA	Excision partielle, lobe du poumon, approche endoscopique [chirurgie thoracique vidéo assistée]
1GR87NW	Excision partielle, lobe du poumon, approche intrapéricardique [transpéricardique]
1GR87PN	Excision partielle, lobe du poumon, télémanipulation robotisée d’outils
1GR87QB	Excision partielle, lobe du poumon, approche thoracique ouverte
1GR89DA	Excision totale, lobe du poumon, approche endoscopique [chirurgie thoracique vidéo assistée]
1GR89NW	Excision totale, lobe du poumon, approche intrapéricardique [transpéricardique]
1GR89QB	Excision totale, lobe du poumon, approche thoracique ouverte
1GR91NW	Excision radicale, lobe du poumon, approche intrapéricardique ouverte [transpéricardique] avec fermeture simple
1GR91NWXXA	Excision radicale, lobe du poumon, approche intrapéricardique ouverte [transpéricardique] avec autogreffe [péricarde]
1GR91NWXXF	Excision radicale, lobe du poumon, approche intrapéricardique ouverte [transpéricardique] avec lambeau libre
1GR91NWXXG	Excision radicale, lobe du poumon, approche intrapéricardique ouverte [transpéricardique] avec lambeau pédiculé à distance
1GR91NWXXL	Excision radicale, lobe du poumon, approche intrapéricardique ouverte [transpéricardique] avec xénogreffe
1GR91NWXXN	Excision radicale, lobe du poumon, approche intrapéricardique ouverte [transpéricardique] avec matériel synthétique
1GR91NWXXQ	Excision radicale, lobe du poumon, approche intrapéricardique ouverte [transpéricardique] avec source combinée de tissus
1GR91QB	Excision radicale, lobe du poumon, approche thoracique ouverte avec fermeture simple
1GR91QBXXA	Excision radicale, lobe du poumon, approche thoracique ouverte avec autogreffe [péricarde]
1GR91QBXXF	Excision radicale, lobe du poumon, approche thoracique ouverte avec lambeau libre
1GR91QBXXG	Excision radicale, lobe du poumon, approche thoracique ouverte avec lambeau pédiculé à distance
1GR91QBXXN	Excision radicale, lobe du poumon, approche thoracique ouverte avec matériel synthétique
1GR91QBXXQ	Excision radicale, lobe du poumon, approche thoracique ouverte avec source combinée de tissus
1GT80LA	Réparation, poumon NCA, approche ouverte
1GT87DA	Excision partielle, poumon NCA, approche endoscopique [chirurgie thoracique vidéo assistée]
1GT87NW	Excision partielle, poumon NCA, approche intrapéricardique [transpéricardique]
1GT87QB	Excision partielle, poumon NCA, approche thoracique ouverte
1GT89DA	Excision totale, poumon NCA, approche endoscopique [chirurgie thoracique vidéo assistée]
1GT89NW	Excision totale, poumon NCA, approche intrapéricardique [transpéricardique]
1GT89QB	Excision totale, poumon NCA, approche thoracique ouverte
1GT91NW	Excision radicale, poumon NCA, avec fermeture simple approche intrapéricardique ouverte [transpéricardique]
1GT91NWXXF	Excision radicale, poumon NCA, avec lambeau libre approche intrapéricardique ouverte [transpéricardique]
1GT91NWXXG	Excision radicale, poumon NCA, avec lambeau pédiculé à distance approche intrapéricardique ouverte [transpéricardique]
1GT91NWXXN	Excision radicale, poumon NCA, avec matériel synthétique approche intrapéricardique ouverte [transpéricardique]
1GT91NWXXQ	Excision radicale, poumon NCA, avec source combinée de tissus approche intrapéricardique ouverte [transpéricardique]
1GT91QB	Excision radicale, poumon NCA, avec fermeture simple approche thoracique ouverte
1GT91QBXXF	Excision radicale, poumon NCA, avec lambeau libre approche thoracique ouverte
1GT91QBXXG	Excision radicale, poumon NCA, avec lambeau pédiculé à distance approche thoracique ouverte
1GT91QBXXN	Excision radicale, poumon NCA, avec matériel synthétique approche thoracique ouverte
1GT91QBXXQ	Excision radicale, poumon NCA, avec source combinée de tissus approche thoracique ouverte
**C.** **Canadian classification of diagnostic, therapeutic, and surgical procedures (CCP) codes**	
**C1. Chemotherapy**	
1355	Injection/infusion d’agents chimiotherap.du cancer/Injection agents chimiothérapeutiques
**C2. Radiotherapy ^2^**	
621	Teleradiotherapie
623	Teleradiotherapie par electrons
624	Teleradiotherapie par autres particules
639	Autre procede radiotherapeutique
**C3. Surgery—Lung Resection**	
4411	Resection en bloc des bronches
4419	Autres excision des bronches nca/Exérèse bronches
4439	Resection segmentaire du poumon (basilaire) (superieur)/Segmentectomie pulmonaire
4449	Lobectomie du poumon/Lobectomie pulmonaire
4459	Pneumonectomie/Pneumonectomie complete
4499	Autre excision du poumon
4543	Autre reparation et plastie des bronches/Réparation et plastie bronches
4545	Autre reparation et plastie du poumon
4593	Aut. op. sur les bronches
4595	Aut. op. sur le poumon
4639	Exérèse lésion cage paroi thoracique/Exc./destruction de lesion de la cage thoracique
4669	Réparation paroi thoracique

^1^ Even though palliative immunotherapy is not indicated for treating lung cancer patients whose tumors harbor an EGFR mutation, the billing code for intravenous infusion of gamma globulins (152) was included in the chemotherapy category to account for situations where it may have been prescribed prior to an EGFR-TKI treatment. ^2^ All radiotherapy codes were used to group patients into 1 of 6 initial treatment code scenarios, however, only the code 8519 (in bold) was used for subsequent steps of the algorithm.

## Appendix C. Literature Review on Experimental and Real-World Studies Reporting Survival Data for EGFR-TKIs for Indications of Interest

A preliminary literature search of the “snowball” type was carried out through the MEDLINE (PubMed) database. The goal of this preliminary search was to explore the literature to develop the search strategy and inclusion and exclusion criteria of our final literature review. PubMed’s “similar articles” function allowed us to consult randomized controlled trials (i.e., experimental trials) on EGFR-TKIs similar to those presented in the summary tables of the algorithm for care of lung cancer patients [69].

The final systematic search of the literature was conducted to include all experimental trials and real-world studies reporting overall survival data related to the three EGFR-TKIs for indications of interest. In May 2021, we searched for articles in French or English that were published since 2005 in the following bibliographic databases: MEDLINE (Ovid), Embase (Ovid) et EBM Reviews: Cochrane Database of Systematic Reviews (EBSCO). Table A5 and Table A6 provide information on the PICO-based (i.e., population, intervention, comparator, outcome) inclusion/exclusion criteria and search strategy, respectively. The studies that were submitted to INESSS for evaluation of the EGFR-TKI for indications of interest and reported overall survival results were included in our review, irrespective of inclusion/exclusion criteria. [20,23,24,25,26,27,28] The results from the article selection process are presented in a flow diagram in Figure A2.

Two reviewers (SQ and GB) were each responsible for a part of the abstract and full-text screening. The references of retained articles were also searched. Data extraction was divided between the two reviewers and particular attention was given to subsequent treatments (i.e., osimertinib) in studies on 1st-line treatment with gefitinib or afatinib. Table A7 provides the data abstracted from each study, in addition to the median overall survival results that are presented in Figure 5 of the main text.

**Table A5:** Inclusion and Exclusion Criteria.

PICO	Criteria	
	Inclusion	Exclusion
**Population (P)**	Patients with advanced stage non-small cell lung cancer and tumours with activating mutation in the EGFR gene For osimertinib: patients with tumors harboring the T790M mutation in the EGFR gene	Main study objectives that may have led to selection bias (e.g., assessment of overall survival in the presence of a biomarker that is measured in a fraction of patients who received the treatment of interest) Overall survival is reported only for subgroups of patients (e.g., treatment-naïve patients, patients in a specific age group)
**Intervention (I)**	Gefitinib as 1st-line palliative treatment Afatinib as 1st-line palliative treatment Osimertinib after the use of a 1st or 2nd generation EGFR-TKI	For the gefitinib and afatinib: the use of osimertinib in a subsequent line (when mentioned) unless it involves less than 5% of all patients.^2^
**Comparator (C)**	No specific comparator is required in experimental studies No comparator is required in real-world studies	-
**Outcomes (O)**	Median overall survival	-
**Study Design**	Experimental trials, real-world studies, systematic reviews ^1^, meta-analyses ^1^	Narrative reviews

^1^ Systematic reviews and meta-analyses were only included to search for additional studies that may have been missed by the literature review. ^2^ Osimertinib is known to increase overall survival relative to standard chemotherapy when given as a subsequent EGFR-TKI [19,20].

**Table A6:** Search Strategy.

MEDLINE (Ovid) Retrieval date: May 2021 Restrictions: 2005-; English, French	
1	(gefitinibOR iressa* OR ZD1839* OR ZD-1839*).ti,ab,hw,kf,kw
2	(afatinib* OR giotrif OR gilotrif OR BIBW-2992* OR BIBW2992*).ti,ab,hw,kf,kw
3	(osimertinib OR tagrisso OR AZD9291* OR AZD-9291*).ti,ab,hw,kf,kw
4	(adenocarcinoma* OR non-squamous OR non-small-cell lung cancer OR NSCLC).ti,ab,hw,kf,kw
5	survival.ti,ab
6	lung.ti,ab
7	1 AND 4 AND 5 AND 6
8	2 AND 4 AND 5 AND 6
9	3 AND 4 AND 5 AND 6
10	(animal* OR cell line* OR vitro OR invitro OR rat OR rats OR mouse OR mice).ti,ab
11	(case report* OR comment* OR reply OR replies OR editorial* OR letter* OR cost* OR econom* OR conference* OR opinion* OR consensus).ti
12	(Case Reports OR Comment OR Editorial OR Letter OR Review OR Guideline OR Practice Guideline OR Consensus Development Conference).pt
13	(Meta-Analysis OR Randomized Controlled Trial OR Systematic Review).pt
14	12 AND 13
15	12 NOT 14
16	7 NOT 10
17	16 NOT 11
18	17 NOT 15
19	(random* OR meta-analy* OR real-world OR real-life OR retrospect* OR observational).ti,ab,hw,kf,kw
20	17 AND 19
21	18 OR 20
22	8 NOT 10
23	22 NOT 11
24	23 NOT 15
25	19 AND 23
26	24 OR 25
27	9 NOT 10
28	27 NOT 11
29	28 NOT 15
30	19 AND 27
31	29 OR 30
**Embase (Ovid)** **Retrieval date: May 2021** **Restrictions: 2005-; English, French**	
1	(gefitinib* OR iressa* OR ZD1839* OR ZD-1839*).ti,ab,
2	(afatinib* OR giotrif OR gilotrif OR BIBW-2992* OR BIBW2992*).ti,ab
3	(osimertinib OR tagrisso OR AZD9291* OR AZD-9291*).ti,ab
4	(adenocarcinoma* OR non-squamous OR non-small-cell lung cancer OR NSCLC).ti,ab
5	survival.ti,ab
6	1 AND 4 AND 5
7	2 AND 4 AND 5
8	3 AND 4 AND 5
9	(animal* OR cell line* OR vitro OR invitro OR rat OR rats OR mouse OR mice).ti,ab
10	(case report* OR comment* OR reply OR replies OR editorial* OR letter* OR cost* OR econom* OR conference* OR opinion* OR consensus).ti
11	(Case Report OR Comment OR Editorial OR Letter OR “Review” OR Practice Guideline OR Consensus Development).pt
12	Meta Analysis/OR Randomized Controlled Trial/OR “Systematic Review”/
13	11 AND 12
14	11 NOT 13
15	6 NOT 9
16	15 NOT 10
17	16 NOT 14
18	(random* OR meta-analy* OR real-world OR real-life OR retrospect* OR observational).ti,ab,hw,kw
19	16 AND 18
20	17 OR 19
21	limit 20 to embase
22	limit 20 to exclude medline journals
23	21 OR 22
24	7 NOT 9
25	24 NOT 10
26	25 NOT 14
27	18 AND 25
28	26 OR 27
29	limit 28 to embase
30	limit 28 to exclude medline journals
31	29 OR 30
32	8 NOT 9
33	32 NOT 10
34	33 NOT 14
35	18 AND 33
36	34 OR 35
37	limit 36 to embase
38	limit 36 to exclude medline journals
39	37 OR 38
**EBM Reviews: Cochrane Database of Systematic Reviews (EBSCO)** **Retrieval date: May 2021** **Restrictions: 2005-; English, French**	
1	(gefitinib* OR iressa* OR ZD1839* OR ZD-1839*).mp
2	(afatinib* OR giotrif OR gilotrif OR BIBW-2992* OR BIBW2992*).mp
3	(osimertinib OR tagrisso OR AZD9291* OR AZD-9291*).mp
4	(adenocarcinoma* OR non-squamous OR non-small-cell lung cancer OR NSCLC).mp
5	survival.mp
6	1 AND 4 AND 5
7	2 AND 4 AND 5
8	3 AND 4 AND 5

**Table A7:** Articles Included for Indirect Comparison of Median Overall Survival Results.

Author(s) and Year [Study Name]	Geographical Region	Single Center/Multicenter	n (Group with EGFR-TKI of Interest)	Median Follow-Up (Months)	Osimertinib as a Subsequent Treatment
1st-line gefitinib treatment					
*Experimental Trials*					
Garcia-Campelo et al., 2020 [29] [GOAL]	Spain and Mexico	Multicenter	91	26.2	−Subsequent treatments not mentioned −End of study follow-up: September 2018
Yoshioka et al., 2019 [30] [WJTOG3405]	Japan	Multicenter	88	59.1	No patient received osimertinib
Yang et al., 2018 [31]	China	Single center	35	N/A	−Subsequent treatments not mentioned −Recruitment until February 2012
Han et al., 2017 [32]	China	Single center	81	N/A	−Subsequent targeted treatment in 3 patients (imprecise on treatment name) −End of study follow-up: October 2016
Patil et al., 2017 [33]	India	Single center	145	14.2	−Subsequent treatments mentioned, osimertinib not mentioned −End of study follow-up: July 2016
Inoue et al., 2013 [26] [NEJ002] ^a^	Japan	Multicenter	114	23.2	No patient could have received osimertinib before the end of study follow-up (December 2010)
Fukuoka et al., 2011 [27] [IPASS] ^a,b^	East Asia	Multicenter	132	17.0	No patient could have received osimertinib before the end of study follow-up (June 2010)
***Real-World Studies***					
Brat et al., 2020^c^ [34]	Czech Republic	Multicenter	325	N/A	−Subsequent treatments not mentioned −End of study follow-up date not mentioned
Hsieh et al., 2020 [35]	Taiwan	Multicenter	3982	20.3	−Subsequent treatments not mentioned −End of study follow-up: December 2016
Choi et al., 2018 [36]	South Korea	Single center	60	18	−Subsequent treatments mentioned, osimertinib not mentioned −End of study follow-up date not mentioned
Imai et al., 2017 [37]	Japan	Multicenter	138	N/A	−Subsequent treatments not mentioned −End of study follow-up date not mentioned
Krawczyk et al., 2017 [38]	Poland	Multicenter	66	N/A	No patient received osimertinib
Sutiman et al., 2017 [39]	Singapore	Single center	383	N/A	−Subsequent treatments not mentioned −End of study follow-up date not mentioned
Inoue et al., 2016 [40]	Japan	Multicenter	929	N/A	−Subsequent treatments not mentioned −Very low chances that patients received osimertinib before the end of study follow-up (December 2014)
Vavala et al., 2016 [41] [BE-POSITIVE]	Italy	Multicenter	288	N/A	−Subsequent treatments mentioned, osimertinib not mentioned −No patient could have received osimertinib before the end of study follow-up (May 2013)
Lai et al., 2014 [42]	Taiwan	Single center	85	N/A	−Subsequent treatments not mentioned −End of study follow-up date not mentioned −Very low chances that patients received osimertinib before publication date
Park et al., 2013 [43]	South Korea	Single center	50	N/A	−Subsequent treatments not mentioned −Very low chances that patients received osimertinib before publication date
**1st-line afatinib treatment**					
***Experimental Trials***					
Yang et al., 2015 ^d^ [28] [LUX-Lung 3] ^e^	International (Asian: 71.7%)	Multicenter	230	41	1% of patients received osimertinib after afatinib (threshold for study exclusion: >5%)
Yang et al., 2015 ^d^ [28] [LUX-Lung 6] ^e^	China, South Korea, and Thailand	Multicenter	242	33	No patient received osimertinib
Yang et al., 2015 ^d^ [28] [LUX-Lung-3 and 6 combined]	International	Multicenter	472	N/A	See individual studies above
***Real-World Studies***					
Brat et al., 2020 ^c^ [34]	Czech Republic	Multicenter	147	N/A	−Subsequent treatments not mentioned −End of study follow-up date not mentioned
Li et al., 2019 [44]	United States	Multicenter	87	12	No patient received osimertinib
Liang et al., 2018 [45]	Taiwan	Single center	259	22	−Subsequent treatments not mentioned −End of study follow-up: August 2017
**Post-EGFR-TKI osimertinib treatment**					
***Experimental Trials***					
Papadimitrakopoulou et al., 2020 [20] [AURA3] ^f^	International	Multicenter	279	23.5	-
***Real-World Studies***					
Chen et al., 2020 [46]	China	Single center	246	42.83	-
Kishikawa et al., 2020 [47]	Japan	Multicenter	56	15.1	-
Lin et al., 2020 [48]	Taiwan	Single center	162	15.8	-
Cao et al., 2019 [49]	China	Single center	74	9	-
Kato et al., 2019 [50]	Japan	Single center	31	12	-

^a^ Study submitted to INESSS for evaluation of gefitinib for enlistment on Quebec’s drug formulary. ^b^ According to the literature review inclusion/exclusion criteria, the IPASS study should have been excluded since the study is restricted to a non-smoking or light smoking population. However, this study was included since all studies submitted to INESSS for drug evaluation were automatically included in the review. ^c^ This article was included twice for overall survival comparisons, once in relation to 1st-line gefitinib treatment and once to 1st-line afatinib treatment. ^d^ This article included overall survival results from two RCTs, including their combined results. ^e^ Study was submitted to INESSS for evaluation of afatinib for enlistment on Quebec’s drug formulary. ^f^ Study was submitted to INESSS for evaluation of osimertinib for enlistment on Quebec’s drug formulary. However, the overall survival results were not mature at the time of submission in the article by Mok and colleagues [19].

## Appendix D. Additional Patient Characteristics

**Table A8:** Characteristics of Patients in Each EGR-TKI Cohort.

Characteristics	1st-Line Gefitinib Cohort ^1^		1st-Line Afatinib Cohort ^1^		Post-EGFR-TKI Osimertinib Cohort ^1^		Lung Cancer Cohort 2016 ^2^	
	n	(%)	n	(%)	n	(%)	n	(%)
**Sex**								
Male	313	(68.5)	48	(60.0)	84	(70.6)	4803	(49.3)
Female	144	(31.5)	32	(40.0)	35	(29.4)	4936	(50.6)
**Age in years**								
Median (range) ^3^	70.6	(34; 93)	68.4	(22; 93)	72.3	(24; 92)	70.7	(NR)
< 50 years	15	(3.3)	4	(5.0)	5	(4.2)	249	(2.6)
50–64 years	91	(19.9)	24	(30.0)	26	(21.8)	2716	(27.9)
65–79 years	271	(59.3)	46	(57.5)	64	(53.8)	4762	(48.8)
≥80	80	(17.5)	6	(7.5)	24	(20.2)	2012	(20.6)
**1st-line EGFR-TKI**								
Gefitinib	457	(100)	-		92	(77.5)	-	
Afatinib	-		80	(100)	25	(21.0)	-	
Erlotinib	-		-		2	(1.7)	-	
**Material Vulnerability Index ^4^**								
0	5	(1.1)	0	(0.0)	1	(0.8)	98	(1.0)
(− vuln.) Quintile 1	82	(17.9)	12	(15.0)	27	(22.7)	1217	(12.5)
Quintile 2	92	(20.1)	12	(15.0)	22	(18.5)	1594	(16.3)
Quintile 3	81	(17.7)	16	(20.0)	22	(18.5)	1916	(19.6)
Quintile 4	88	(19.3)	19	(23.8)	15	(12.6)	2288	(23.5)
(+ vuln.) Quintile 5	107	(23.4)	18	(22.5)	31	(26.1)	2473	(25.4)
Missing	2	(0.4)	3	(3.8)	1	(0.8)	166	(1.7)
**Number of comorbidities ^5^**								
Median	6	NR	7	(NR)	6	(NR)	NR	(NR)
0–4	169	(37.0)	27	(33.8)	42	(35.3)	NR	(NR)
5–9	160	(35.0)	30	(37.5)	50	(42.0)	NR	(NR)
10–14	80	(17.5)	17	(21.3)	19	(16.0)	NR	(NR)
15–19	34	(7.4)	4	(5.0)	8	(6.7)	NR	(NR)
≥ 20	13	(2.8)	2	(2.5)	0	(0)	NR	(NR)
N/AV	1	(0.2)	0	(0)	0	(0)	NR	(NR)
**Total**	457		80		119		9752	

Abbreviations: NR: not reported. ^1^ Age measured at the start of the EGFR-TKI of interest. ^2^ Quebec lung cancer cohort developed by INESSS, in which age was measured at the diagnosis date [3]. ^3^ Range: minimum; maximum. ^4^ Patients’ dissemination areas (DA: smallest Canada-wide geographic unit with a standard population size of 400–700) were obtained from the health insurance registry (FIPA) and linked to a DA-level material vulnerability index that was developed at INESSS with the 2016 Census data (DA-level unemployment ratio, median income, and low education). The index was adapted from a previous method and quintiles were created based on the total RAMQ population (Q1: least deprived and Q5: most deprived [70]. ^5^ The Population Grouping Methodology was applied to obtain the total number of comorbidities for each patient [71,72]. Patients’ hospitalization data was screened for diagnostic codes for 226 possible health conditions (excluding lung cancer) in the three years prior to patients’ 1st EGFR-TKI.
